# The complete chloroplast genome sequence of *Monotropa uniflora* (Ericaceae)

**DOI:** 10.1080/23802359.2020.1806754

**Published:** 2020-08-14

**Authors:** XueDie Liu, XingYu Liao, DeQiang Chen, Yu Zheng, Xia Yu, XinYu Xu, ZhongJian Liu, SiRen Lan

**Affiliations:** a College of Forestry, Fujian Agriculture and Forestry University, Fuzhou, China; b Key Laboratory of National Forestry and Grassland Administration for Orchid Conservation and Utilization at College of Landscape, Fujian Agriculture and Forestry University, Fuzhou, China

**Keywords:** *Monotropa uniflora*, plastid genome, phylogenetic analysis, Ericaceae

## Abstract

*Monotropa uniflora* is one of the representative plants of Ericaceae family, which was famous for entire translucent and ‘ghostly’ white. Also, unique lifestyle also attracts lots of researchers, which it obtains through fixed carbon from photosynthetic plants via a shared mycorrhizal network. In this study, the complete chloroplast (cp) genome of *M. uniflora* was assembled and annotated, its full-length is 26,913 bp. Plastid genome contains 31 genes, 14 protein-coding genes, 14 tRNA genes, and 3 rRNA genes. The phylogenetic analyses based on the complete chloroplast genome sequence provided solid evidence that *M. uniflora* has a close relationship *M. odorata.* The chloroplast genome presented here will help for further conservation of *M. uniflora* and other saprophytes.

Ericaceae is a large and diverse family of eudicots comprising approximately 120 genera and is divided into seven subfamilies(Kron et al. 2002; Logacheva et al. 2016). Many species of this family have a great economic or ornamental value. Most members of Ericaceae form associations with mycorrhizal fungi, on which they are dependent for such processes as nitrogen and phosphorus acquisition (Lutz and Sjolund 1973; Read and Stribley 1973). *Monotropa uniflora* is one of the representative plants of Ericaceae family. The entire plant is ‘ghostly’ white or sometimes pale pinkish-white, and commonly has black flecks (Kron et al. 2002). The leaves are scale-like and flecked with black on the flower stalk (peduncle). Furthermore, this dependence on fungi is most notable in species from the subfamily Monotropoidae, the members of which are partially or completely mycoheterotrophic, obtaining carbon through fungal interactions (Logacheva et al. 2016). It is urgent to establish an effective conservation strategy for this significant plant. The complete chloroplast genomic data will be useful for population and phylogenetic studies of *M. uniflora.*

Here, we assembled the complete cp genome of *M. uniflora*. Fresh tissue of *M. uniflora* was acquired from JianOu (118°32′E, 27°05′N), NanPing City, Fujian Province of China, and voucher specimen deposited at Herbarium of College of Forestry, Fujian Agriculture and Forestry University (specimen code FAFU0910). DNA extraction from fresh leaf tissue, with 350 bp randomly interrupted by the Covaris ultrasonic breaker for library construction. The constructed library was sequenced PE150 by Illumina Hiseq Xten platform, approximately 2GB data generated. Illumina data were filtered by script in the cluster (default parameter: -L 5, -p 0.5, -N 0.1). Plastid genome assembled by GetOrganelle pipe-line (https://github.com/Kinggerm/GetOrganelle), it can get the plastid-like reads, and the reads were viewed and edited by Bandage (Wick et al. 2015). Assembled chloroplast genome annotation based on comparison with *M. uniflora* by GENEIOUS R11.15 (Biomatters Ltd., Auckland, New Zealand) (Kearse et al. 2012). The annotation result was draw with the online tool OGDRAW (http://ogdraw.mpimp-golm.mpg.de/) (Lohse et al. 2013).

Recent characterization of the plastid genomes of the photosynthetic Ericaceae revealed many unique shared traits, such as a high repeat content, a drastic reduction in small single-copy regions, the putative pseudogenization of several essential genes and multiple rearrangements (Fajardo et al. 2013; Martínez-Alberola et al. 2013). The complete plastid genome sequence of *M. uniflora* (GenBank accession MT491729) was 26,913 bp in length. As the plastid genome of *M. uniflor* was highly reduced, it was meaningless to distinguish a lager single-copy (LSC), a small single-copy (SSC) and a pair of inverted repeats (IR) regions. In addition, a total of 31 gene species were annotated, including 14 protein-coding (PCG), 14 transfer RNA (tRNA), and 3 ribosomal RNA (rRNA) gene species. The complete genome GC content was 27.10%. In order to reveal the phylogenetic position of *M. uniflora*, a phylogenetic analysis was performed based on 6 complete cp genomes of Ericaceae (*M. uniflora, Monotropa hypopitys*, *Pityopus californicus*, *Hemitomes congestum*, *Allotropa virgate*, *Monotropsis odorata*) and one taxa (*Vaccinium macrocarpon*) as outgroup, all of them were downloaded from NCBI GenBank. The sequences were aligned by HomBlocks (Bi et al. 2018), and phylogenetic tree was constructed by RAxML (Figure 1) (Stamatakis 2014). The relationship between *Monotropa* (*M. uniflora*) and *Monotropsis* (*M. odorata*) was strongly supported (bootstrap = 100%), and the maximum likelihood phylogenetic analysis showed that *M. uniflora*s was sister to *M. odorata.*

**Figure 1.**
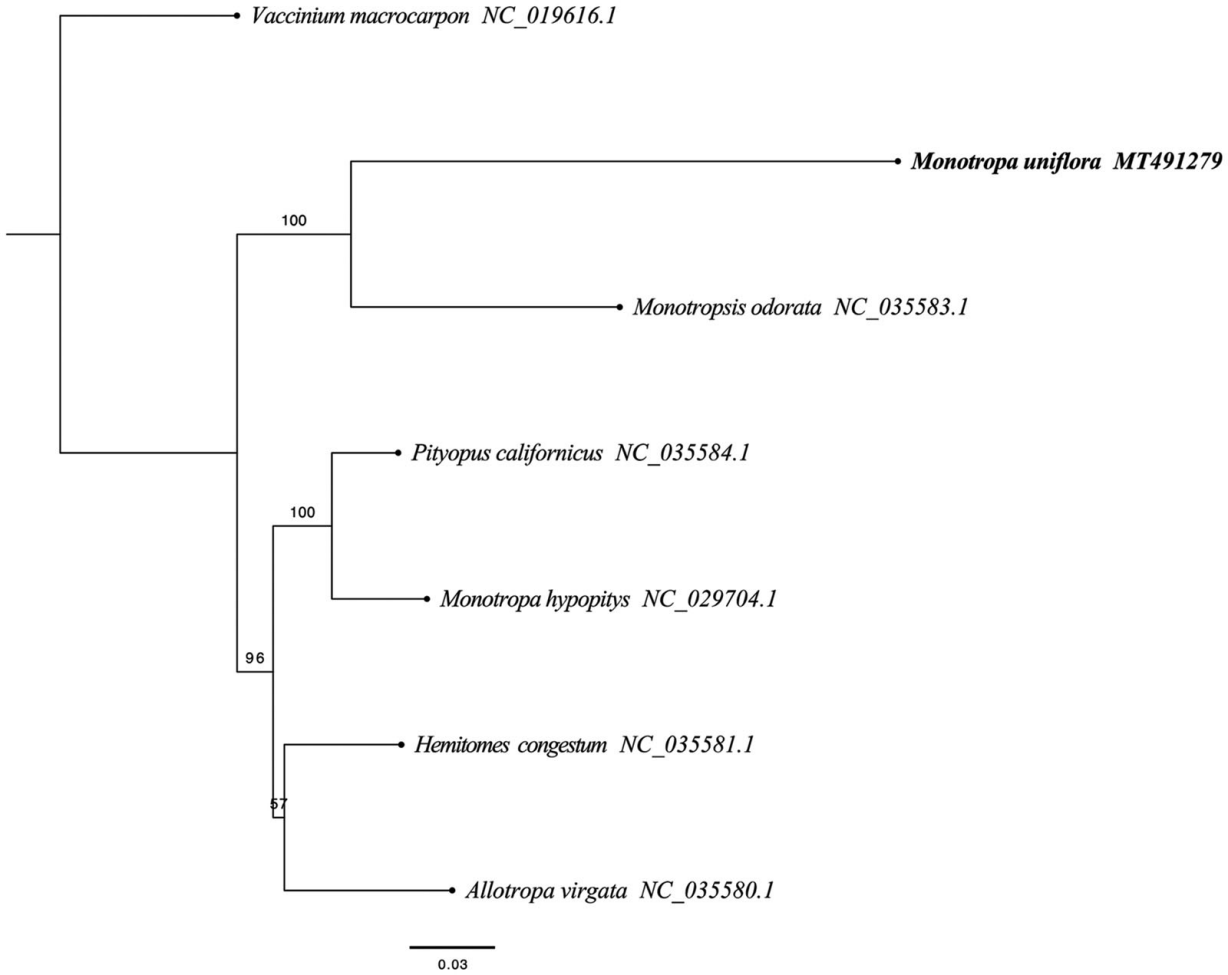
RAxML phylogeny of *M. uniflora* based on 7 complete cp genomes (Bootstrap values are shown above each node).

## Data Availability

The data that support the findings of this study are openly available in GenBank of NCBI at https://www.ncbi.nlm.nih.gov/, reference number MT491729.
